# HIF1α-Induced by Lysophosphatidic Acid Is Stabilized via Interaction with MIF and CSN5

**DOI:** 10.1371/journal.pone.0137513

**Published:** 2015-09-09

**Authors:** Yi Ran No, Sei-Jung Lee, Ajay Kumar, C. Chris Yun

**Affiliations:** 1 Division of Digestive Diseases, Department of Medicine, Emory University, Atlanta, Georgia, United States of America; 2 Winship Cancer Institute, Emory University, Atlanta, Georgia, United States of America; University of Virginia, UNITED STATES

## Abstract

Macrophage migration inhibitory factor (MIF) is a cytokine that has broad effects on immune system and inflammatory response. A growing body of evidence implicates the role of MIF in tumor growth and metastasis. Lysophosphatidic acid (LPA), a bioactive lipid mediator, regulates colon cancer cell proliferation, invasion, and survival through LPA_2_ receptor. Loss of LPA_2_ results in decreased expression of MIF in a rodent model of colon cancer, but the mechanism of MIF regulation by LPA is yet to be determined. In this study, we show that LPA transcriptionally regulates MIF expression in colon cancer cells. MIF knockdown decreased LPA-mediated proliferation of HCT116 human adenocarcinoma cells without altering the basal proliferation rates. Conversely, extracellular recombinant MIF stimulated cell proliferation, suggesting that the effect of MIF may in part be mediated through activation of surface receptor. We have shown recently that LPA increases hypoxia-inducible factor 1α (HIF1α) expression. We found that MIF regulation by LPA was ablated by knockdown of HIF1α, indicating that MIF is a transcriptional target of HIF1α. Conversely, knockdown of MIF ablated an increase in HIF1α expression in LPA-treated cells, suggesting a reciprocal relationship between HIF1α and MIF. LPA stimulated co-immunoprecipitation of HIF1α and MIF, indicating that their association is necessary for stabilization of HIF1α. It has been shown previously that CSN9 signalosome subunit 5 (CSN5) interacts with HIF1α to stabilize HIF1α under aerobic conditions. We found that LPA did not alter expression of CSN5, but stimulated its interaction with HIF1α and MIF. Depletion of CSN5 mitigated the association between HIF1α and MIF, indicating that CSN5 acts as a physical link. We suggest that HIF1α, MIF, and CSN5 form a ternary complex whose formation is necessary to prevent degradation of HIF1α under aerobic conditions.

## Introduction

Macrophage migration inhibitory factor (MIF) was originally identified as a product of activated T cells, but it is now recognized as a chemokine that plays a central role in innate and adaptive immunity [1]. Through its pro-inflammatory effects, MIF has been implicated in the pathogenesis of several acute and chronic inflammatory conditions, including rheumatoid arthritis, atherosclerosis, and septic shock [2]. MIF is expressed by a variety of cells including endothelial cells, mesenchymal cells, eosinophils, and epithelial cells. In the intestinal tract, MIF is expressed primarily by epithelial cells, also by a poorly characterized lamina propria cell population [3]. Polymorphism of MIF gene has been linked to the susceptibility to inflammatory bowel diseases [4, 5]. Inhibition or loss of MIF protects mice from chemically induced colitis, while transgenic MIF expression exacerbates colitic conditions [3, 6, 7].

Unlike typical cytokines, MIF has a tautomerase activity [8]. The crystal structure analysis shows that the active form of MIF consists of a homotrimer, with the tautomerase active sites at the monomer interface [9]. Cytokines usually signal through receptors located on the plasma membrane of a target cell and MIF is no exception in this aspect. MIF is shown to bind CD74, CXCR2, and CXCR4 to induce chemokine responses of monocytes and T cells [10, 11]. The region encompassing the tautomerization site also makes critical contact with the CD74 receptor such that covalent modification of proline at the tautomerization site abolishes tautomerase activity and impairs CD74 binding [12].

Over-expression of MIF has been shown in several neoplasms and expression levels have been found to correlate with disease severity [13–17]. Multiple effects have been ascribed to MIF, including tumor invasion, angiogenesis, and down-regulation of the tumor suppressor p53 [18–20]. Its effect on p53 suggests that increased expression of MIF might exacerbate tumor progression by suppressing p53-mediated growth arrest and apoptosis [18–20]. Increased MIF expression is also observed in human colorectal adenomas, and MIF deficiency reduces tumor incidence and angiogenesis in the *Apc* ^*Min/+*^ model of colon cancer, providing direct evidence for its role in colon carcinogenesis [18].

LPA is a pleiotropic lipid molecule, which mediates a variety of biological effects altering cell growth, motility, survival, and inflammatory responses through a family of G protein-coupled receptors, LPA_1-6_ [21]. A body of evidence provides a linkage between LPA and the pathological progress of cancer [22, 23]. In vivo evidence for the critical importance of LPA_2_ in colon cancer has been demonstrated in the rodent models of *Apc* ^*Min/+*^ and colitis-induced colon cancer, where loss of LPA_2_ reduces tumor burden [24, 25]. Colitis-induced colon cancer in LPA_2_-null mice is associated with a marked decrease in cyclooxygenase-2, monocyte chemoattractant protein 1, and MIF [25]. A previous study has reported that LPA induces MIF in CT26 rodent colon cancer cells [19]. However, how LPA regulates MIF has not been reported. The goal of this study is to determine the mechanism of MIF induction by LPA and to determine the role of MIF in LPA-mediated effects.

## Materials and Methods

### Cell Culture and Plasmids

HCT116, LoVo, HT-29, and SW480 cells were obtained from ATCC and grown as previously described [26]. pLKO.1 plasmids harboring short hairpin RNA (shRNA) targeting LPA_2_ (shLPA_2_), HIF1α (shHIF1α), MIF (shMIF), or CSN5 (shCSN5) were from Sigma. pLKO.1-puro with non-target shRNA was used to generate control lentivirus (shCont). Stable transduced cells were selected using puromycin and pooled cells were used unless otherwise specified. Human MIF cDNA was amplified from HCT116 cDNA using primer pair, F: 5’- TAG CTA GCA TGC CGA TGT TCA TCG TAA ACA -3’ and R: 5’- CAG GAT CCT TAC TTG TCA TCG TCA TCC TTG TAA TCC TTG TCA TCG TCA TCC TTG TAA TCG GCG AAG GTG GAG TTG TTC CAG -3’, to generate MIF with double FLAG tags at the C-terminus, which was subcloned into pcDNA-Hygro, resulting in pcDNA-hygro/MIF-2xFLAG. HA-HIF1α-pcDNA3 was a gift from Dr. William Kaelin (Addgene plasmid #18949) [27]. Cells transfected with pcDNA-hygro/MIF-2xFLAG using lipofectamin 2000 (Life Technologies, Grand Island, NY) were selected using hygromycin. The resulting HCT116/MIF-2xFLAG cells were transiently transfected with HA-HIF1α-pcDNA3 where indicated.

### Chemicals and antibodies

LPA (18:1) was purchased from Avanti Polar Lipids (Alabaster, AL). LPA was used at the final concentration of 1 μM in PBS containing 0.1% fatty acid-free bovine serum albumin (BSA) unless otherwise specified. The same volume of PBS containing 0.1% BSA was used as a control. The following antibodies were purchased: mouse anti-HIF1α (BD Biosciences, Franklin Lakes, NJ); rabbit anti-MIF, mouse anti-FLAG, and mouse anti-actin (Sigma-Aldrich, St. Louis, MO); mouse anti-HA (Covance, Princeton, NJ). Human recombinant MIF (rMIF) was from R&D System (Minneapolis, MN). MIF antagonist, (*S*,*R*)-3-(4-hydroxyphenyl)-4,5-dihydro-5-isoxazole acetic acid methyl ester (ISO-1) was obtained from EMD Millipore (Billerica, MA). All other chemicals and reagents were purchased from Sigma.

### Cell proliferation

Cells were seeded and synchronized by serum starvation for 36 h. Cells were treated with 1μM LPA once a day for up to 3 days and the number of cells were counted daily using a hemocytometer.

*Agarose colony-forming assays*: Cells were suspended in McCoy’s 5A Medium containing 10% delipidated FBS (Atlanta Biologicals) and 0.4% agarose, and plated in triplicate on a 0.7% agarose base in 96-well plates (4,000 cells/well). Cells were then placed in a 37°C and 5% CO2 incubator and allowed to grow under standard conditions for 4 weeks. Dishes were stained with 0.05% crystal violet overnight at 4°C and colonies with a diameter greater than 20 μm were counted in the entire dish under a microscope (Nikon Eclipse TE300).

### ELISA for detection of secreted MIF

Overnight serum-starved cells were treated with LPA for up to 24h. To determine the amount of MIF protein secreted by the cells, conditioned media was collected and centrifuged at 5,000xg for 5 min to remove cell debris. The collected samples were either used for MIF measurement by ELISA (Life Technologies) or frozen at -80°C until needed.

### Western immunoblot and immunoprecipitation

Immunoprecipitation was performed as previously described with a modification [28]. Briefly, Cell treated with LPA or PBS as control were washed twice in cold PBS, scraped, and lysed in 1x Cell Lysis Buffer (Cell Signaling, Danver, CO) containing protease inhibitors (Roche, Indianapolis, IN). The crude lysate was sonicated for 2 × 15 s and spun at 14,000 × *g* for 15 min. Protein concentration was determined by the Bicinchoninic Acid Assay (Sigma). Lysate (300 μg) was pre-cleared by incubation with 30 μl of protein A-Sepharose beads for 1 h and the supernatant was then incubated overnight with primary antibody. Immunocomplex was purified by incubating with 50 μl of protein A-Sepharose beads for 1.5 h, followed by 3 washes in lysis buffer and 2 washes in PBS. All the above steps were performed at 4°C or on ice. The bound immunocomplex was eluted by incubating the protein A beads in Laemmli sample buffer for 10 min at 95°C and were then separated by SDS-PAGE. The proteins were then transferred to nitrocellulose membrane for Western immunoblotting as previously described [28].

*Quantitative RT-PCR (qRT-PCR)*: Total RNA was isolated from cells using TRIzol (Invitrogen), and cDNA was synthesized using the First Strand Synthesis kit (Invitrogen). qRT-PCR was performed as described [24]. The following primer pairs were used: MIF: 5’-GTTCCTCTCCGAGCTCACC-3’ and 5’-TGCTGTAGCGGTTCTG-3’; HIF1α: 5’-CACTACCACTGCCACCACTG-3’ and 5’-CCTTTTCCTGCTCTGTTTGG-3’; CSN5: 5’-TGGGTCTGATGCTAGGAAAGG-3’ and 5’-CTATGATACCACCCGATTGCATT-3’; c-Jun: 5’-TCC AAG TGC CGA AAA AGG AAG-3’ and 5’-CGA GTT CTG AGC TTT CAA GGT-3’; Glut1: 5’-ATT GGC TCC GGT ATC GTC AAC-3’ and 5’-GCT CAG ATA GGA CAT CCA GGG TA-3’; VEGFA: 5’-AGG GCA GAA TCA TCA CGA AGT-3’ and 5’-AGG GTC TCG ATT GGA TGG CA-3’; β-actin: 5’-TCCCTGGAGAAGAGCTACGA-3’ and 5’-AGCACTGTGTTGGCGTACAG-3.

### Confocal immunofluorescence microscopy

Confocal immunofluorescence labeling of HCT116 cells was performed as described [28]. Briefly, cells were washed twice with cold PBS, fixed in 4% paraformaldehyde in PBS for 10 min at room temperature, permeabilized in 0.2% Triton X-100 in PBS for 5 min, and blocked in PBS containing 5% normal goat serum for 30 min at RT. Cells were then stained with primary antibody for overnight at 4°C. Following three washes, 10 min each, with PBS, the cells were incubated with Alexa 488-conjugated donkey anti-mouse IgG or Alexa 555-conjugated goat anti-rabbit IgG (Invitrogen) for 1 h at room temperature. After 3 × 10 min washes with PBS, cells were mounted with ProLong Gold Antifade Reagent (Invitrogen) and observed under a Zeiss LSM510 laser confocal microscope.

*Statistical Analysis*: Data are expressed as means ± standard error of the means (SEM). Statistical significance was determined by a 2-tailed unpaired Student’s *t* test or ANOVA. A *p* value of <0.05 was considered significant.

## Results

### LPA-induced MIF expression is dependent on HIF1α

**Fig 1:**
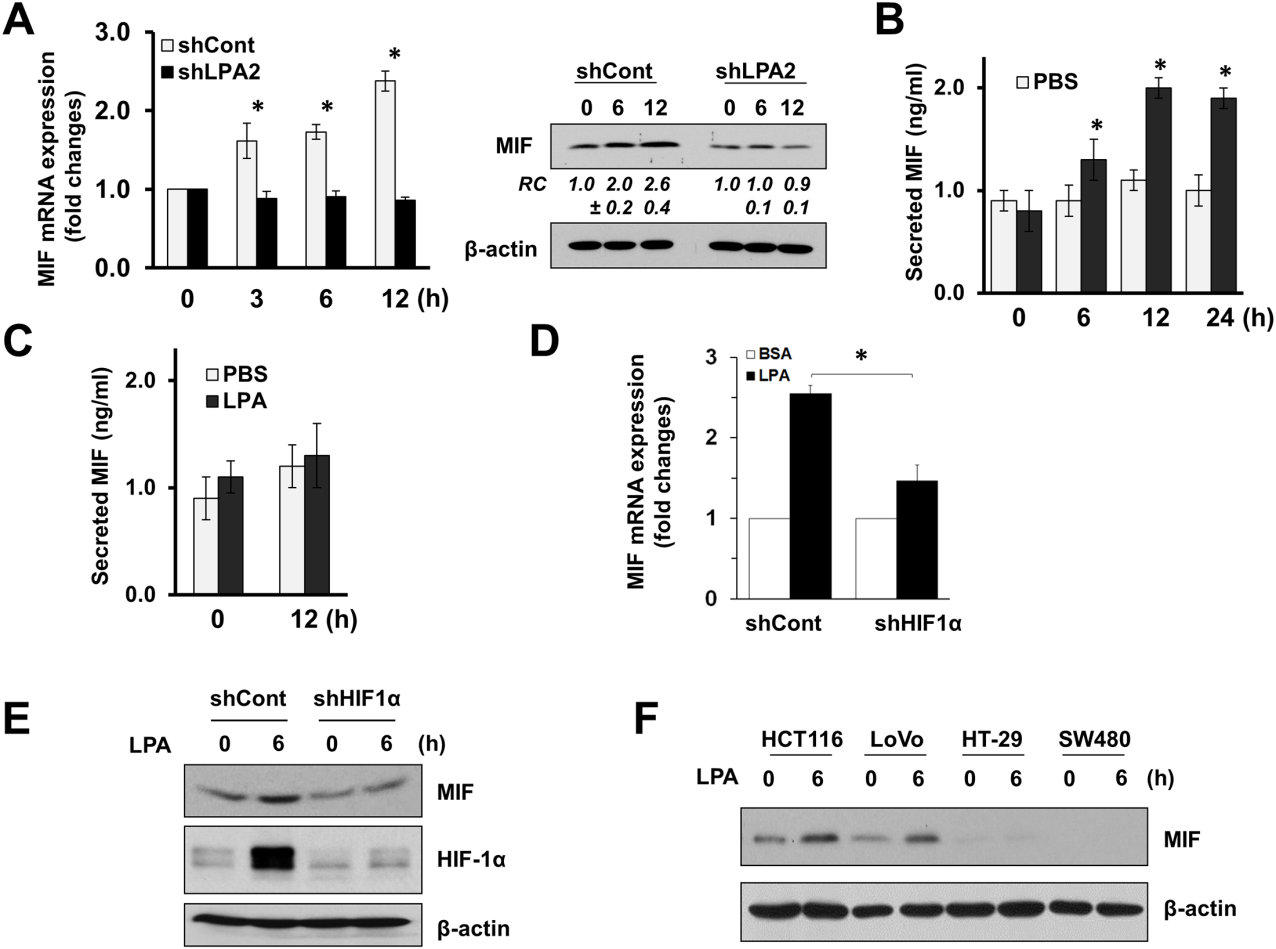
LPA induces MIF in a HIF1α dependent mechanism. **(A)** MIF mRNA (left) and protein (right) expression was determined in HCT116 cells stably transduced with lentiviral shCont and shLPA_2_. Cells were treated with 1 μM LPA for up to 12 h. n = 3. *, *p* < 0.01 compared with untreated cells. RC (mean ± SEM), relative changes in MIF expression quantified by densitometry analysis. **(B)** HCT116 cells treated with PBS or LPA, and MIF secreted into the media was determined by ELISA. n = 3. *, *p* < 0.01 compared with control treated cells. **(C)** MIF secretion by LPA in cells transduced with shLPA_2_ was determined. ns, not significant. HCT116 cells transduced with shCont or shHIF1α were treated with LPA, and the expression levels of MIF mRNA (**D**) and protein (**E**) were determined. *, *p* < 0.01. **(F)** Regulation of MIF by LPA in HCT116, LoVo, HT-29, and SW480 cells is shown. Representative Western blot figures from 3 independent experiments are shown.

We have reported previously that loss of LPA_2_ decreased MIF expression in mouse intestinal mucosa [25]. To determine whether the LPA-LPA_2_ axis is directly responsible for MIF expression, we determined MIF expression in human colon cancer HCT116 cells. LPA increased MIF mRNA and protein expression in HCT116 cells in a time-dependent manner (Fig 1A). The induction of MIF in HCT116 cells was LPA_2_ dependent since knockdown of LPA_2_ completely blocked the increase in MIF expression, consistent with an earlier finding that MIF expression is decreased in LPA_2_-null mice [25]. Moreover, we were able to detect MIF in the media of LPA-treated cells (Fig 1B), indicating that MIF produced is secreted. As expected from data in Fig 1A, LPA-mediated MIF secretion was abolished by knockdown of LPA_2_. [25]

MIF is a known target of HIF1α, and we have shown recently that LPA induces HIF1α in colon cancer cells under normoxic conditions [26, 29]. To investigate the potential contribution of HIF1α in regulation of MIF by LPA, we assessed the effect of HIF1α knockdown on MIF expression. LPA-evoked MIF mRNA and protein expression in HCT116 cells was reduced upon HIF1α knockdown (Fig 1D and 1E), indicating HIF1α dependence. Our previous study showed correlation between LPA-mediated induction of HIF1α and the presence of wild type p53 [26]. We observed an increase in MIF expression in HCT116 and LoVo cells, which harbor wild type p53. In comparison, the basal expression levels of MIF in mutant p53-expressing HT-19 and SW480 cells were low and, importantly, the changes in MIF expression in these cells were hardly detectable (Fig 1F). Together, these results suggest that LPA induces MIF expression and this regulation is HIF1α-dependent.

### MIF stimulates colon cancer cell proliferation

**Fig 2:**
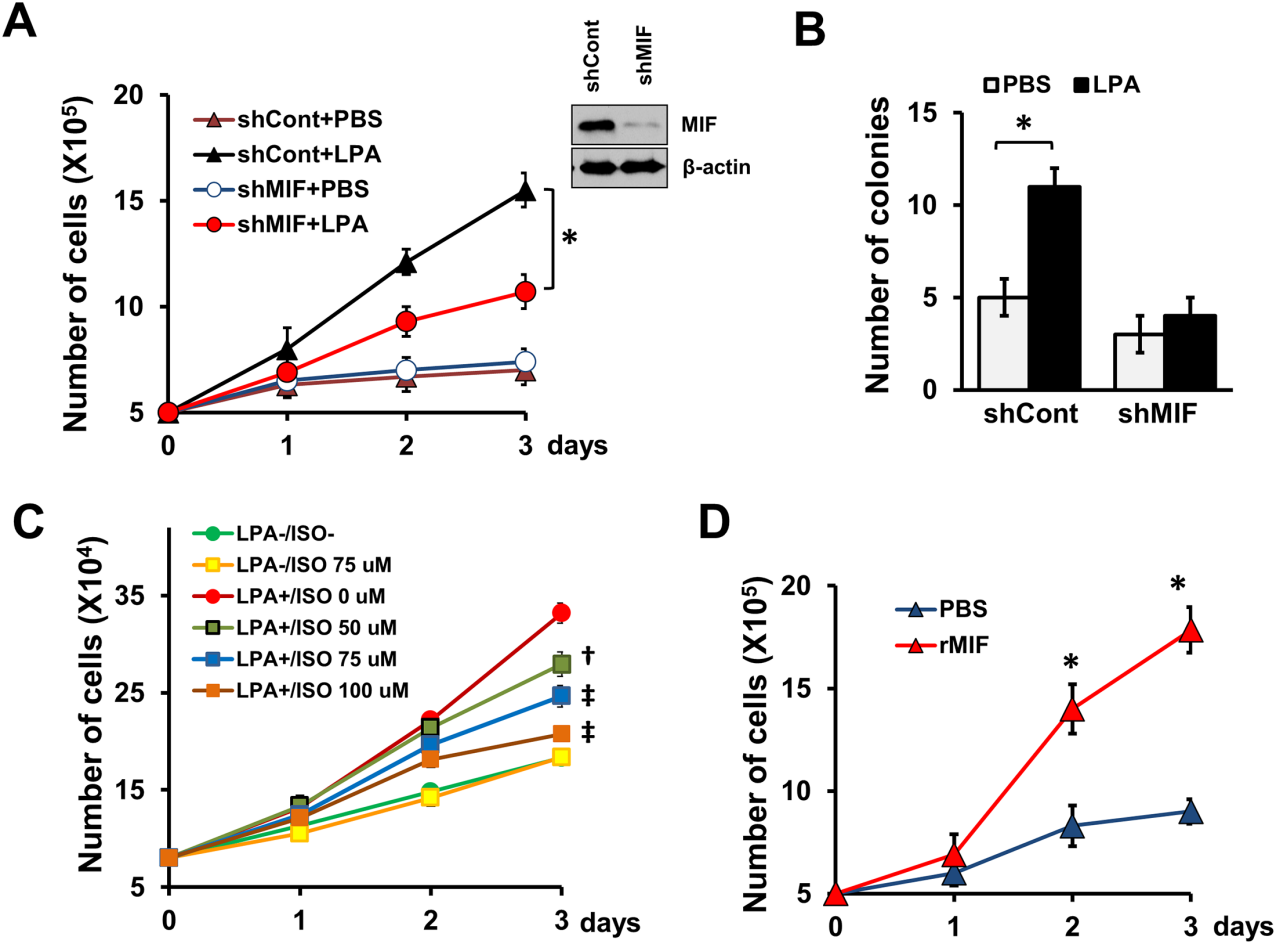
MIF is necessary for LPA-induced cell proliferation and colony formation. **(A)** HCT116 cells transduced with shMIF or shCont were cultured for up to 3 days with daily addition of LPA. Numbers of cells were counted daily. Data (mean ± SEM) are from three independent experiments in triplicates. *, *p* < 0.01 versus shCont+LPA. Western blot shows knockdown of MIF (90%). **(B)** HCT116 cells transduced with shCont or shMIF were seeded in soft-agar and treated with LPA or PBS. Colony numbers (mean ± SEM) were counted as described in the *Materials and Methods*. *, *p* < 0.01. **(C)** Proliferation of HCT116 cells was determined in the presence of different concentrations of ISO-1. Cell numbers were counted as described above. †, *p* < 0.05, ‡, *p* < 0.005 versus cells treated with LPA alone. **(D)** HCT116 cells were cultured for up to 3 days with daily addition of recombinant MIF (rMIF, 100 nM). Numbers of cells were counted daily. Data are from three independent experiments in triplicates. *, *p* < 0.01 versus PBS.

We have shown previously that HIF1α is critical for aerobic growth of colon cancer cells [26]. Because we found HIF1α to be crucial for LPA-induced MIF, we evaluated whether MIF has a role in cell proliferation. Fig 2A and 2B show that knockdown of MIF significantly attenuated LPA-dependent proliferation and anchorage-independent growth of HCT116 cells, respectively. However, the proliferation rate of the unstimulated cells was not impacted by the MIF knockdown. The effect of MIF knockdown was corroborated by using the MIF inhibitor, ISO-1. As shown in Fig 2C, ISO-1 abrogated LPA-dependent proliferation of HCT116 cells in a concentration-dependent manner. MIF exerts its effect in part through surface receptors such as CD74 or CXCR4 [10, 11]. To determine whether extracellular MIF is sufficient to modulate cell proliferation, we evaluated the effect of recombinant MIF (rMIF) added to the media. Fig 2D shows that extracellular rMIF facilitated proliferation of HCT116 cells, indicating that extracellular MIF alone is sufficient to facilitate cell proliferation.

### MIF reciprocally regulates HIF1α

**Fig 3:**
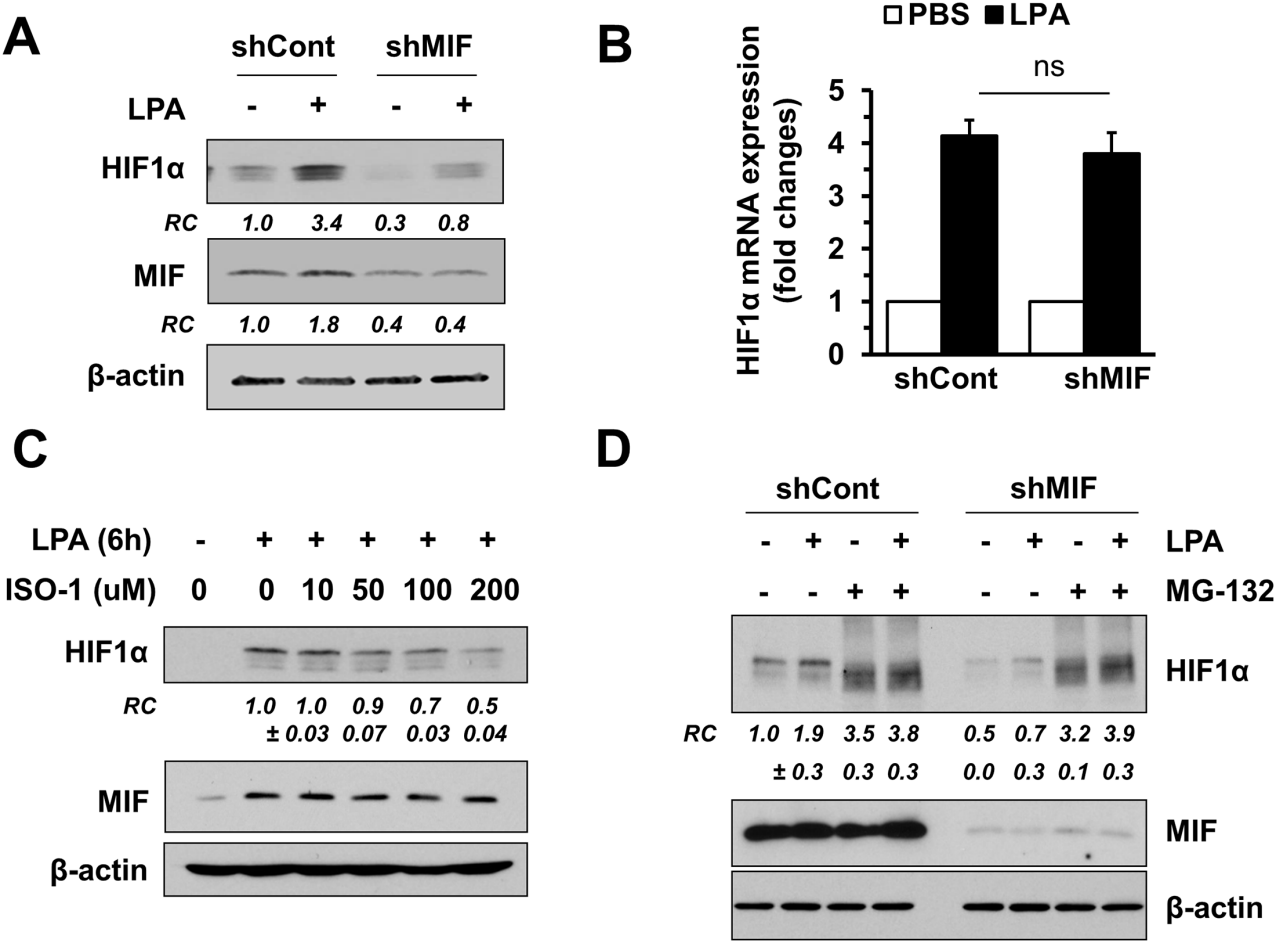
MIF is necessary for the stabilization of HIF1α. The effect of MIF knockdown on HIF1α protein **(A)** and mRNA **(B)** expression was determined. HCT116/shCont and HCT116/shMIF cells were treated with LPA for 6 h, and cells were lysed for protein or RNA. RC (mean ± SEM), relative MIF protein expression quantified by densitometry analysis. ns, not significant. **(C)** Cells were treated with LPA in the presence of increasing concentrations of ISO-1, and HIF1α protein expression was determined. **(D)** HCT116/shCont and HCT116/shMIF cells were pretreated with 10 μM MG132 (+) or DMSO (-) for 3 h prior to treatment with LPA for 3 h. HIF1α and MIF expression is shown. RC (mean ± SEM), relative HIF1α expression quantified by densitometry analysis. Representative Western blot figures from 4 independent experiments are shown in all cases.

Previous studies have shown that MIF stabilizes HIF1α under hypoxic conditions [30, 31]. To have a better understanding of the relationship between HIF1α and MIF in the context of LPA, we determined whether MIF reciprocally affects HIF1α expression. Depletion of MIF significantly blocked LPA-dependent HIF1α protein expression (Fig 3A). However, MIF knockdown did not alter HIF1α mRNA levels, suggesting that the effect of MIF on HIF1α is post-transcriptional (Fig 3B). The importance of MIF in HIF1α induction was further examined by using ISO-1. ISO-1 decreased HIF1α expression in a concentration dependent manner (Fig 3C), demonstrating that blocking the tautomerase active site of MIF interferes with the stabilization of HIF1α. To investigate whether HIF1α is predisposed to degradation in the absence of MIF, we determined the effect of MIF knockdown in the presence of MG-132, a proteasome inhibitor. Fig 3D shows that inhibiting proteasomes by MG-132 restored HIF1α expression in cells transduced with shMIF (7^th^ lane vs 8^th^ lane), suggesting that MIF protects HIF1α from proteasomal degradation.

**Fig 4:**
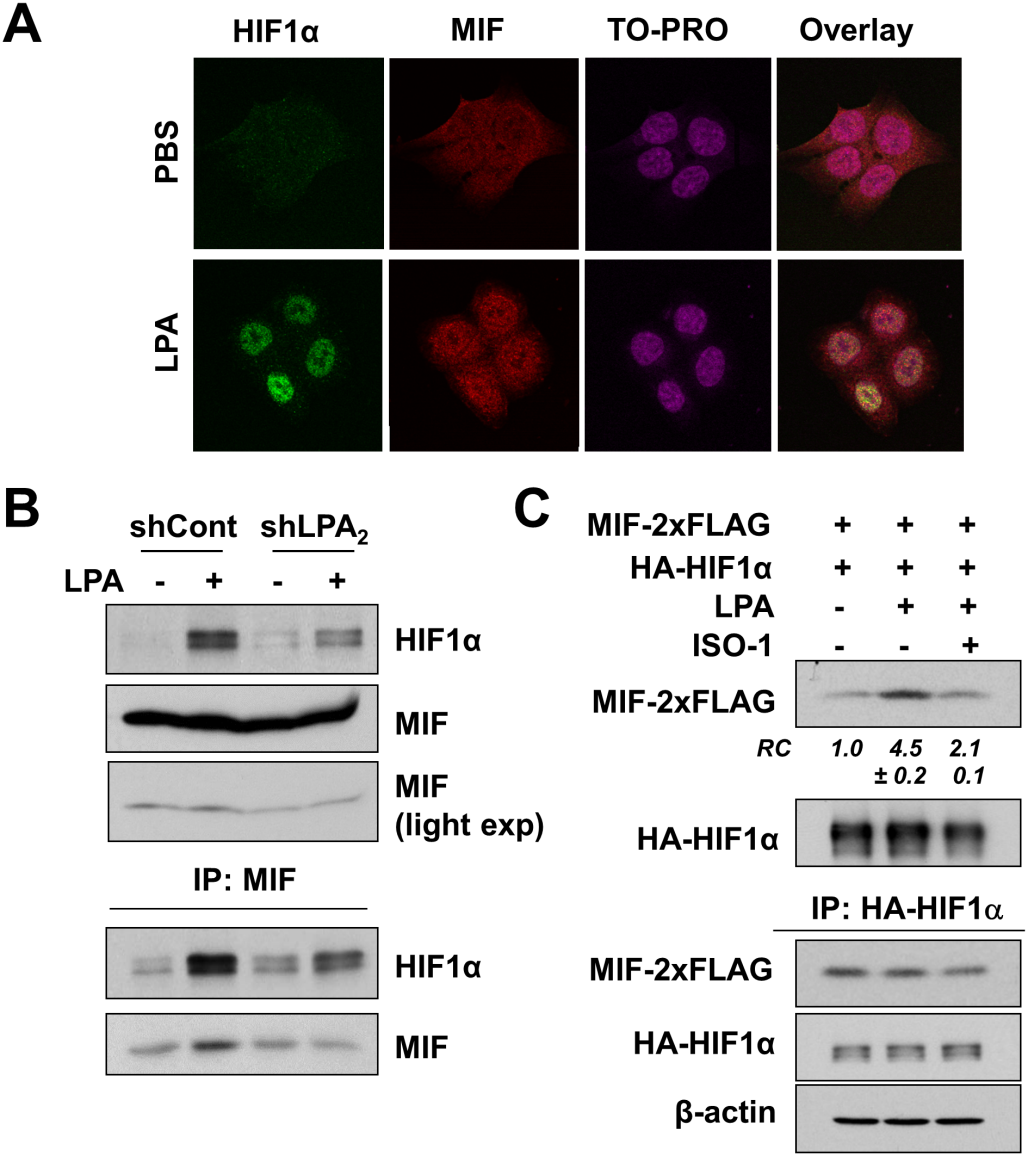
MIF interacts with HIF1α. **(A)** Cellular localization of HIF1α (green) and MIF (red) in cells treated with LPA was determined by immunofluorescence confocal microscopy. TO-PRO was used for nuclear counterstaining (blue). n = 3. **(B)** Co-immunoprecipitation of MIF and HIF1α is shown. HCT116/shCont and HCT116/shLPA_2_ cells were treated with LPA and MIF was immunoprecipitated with anti-MIF antibody (0.5 μg), followed by immunoblotting with anti-HIF1α antibody. Upper panels show HIF1α and MIF in the immunocomplex. Lighter exposure shows that similar amounts of MIF were immunoprecipitated under all conditions. Lower panels show HIF1α and MIF expression in cell lysates. **(C)** HA-HIF1α was transiently expressed in HCT116 cells stably expressing MIF-2xFLAG. Co-immunoprecipitation of HA-HIF1α and MIF-2xFLAG was performed in cells treated with PBS or LPA. To determine the role of MIF, cells were treated with LPA in the presence of 100 mM ISO-1 (3^rd^ lane). Upper panels show MIF-2xFLAG and HA-HIF1α in the immunocomplex. RC (mean ± SEM), relative changes in MIF-2xFLAG quantified by densitometry analysis. Lower panels show MIF-2xFLAG and HA-HIF1α in cell lysates. Representative figures of three independent experiments are shown in all studies.

To investigate whether HIF1α and MIF are spatially associated, we determined the cellular localization of HIF1α and MIF by immunofluorescence analysis. In untreated cells, HIF1α expression was low, while MIF was diffusely expressed in the nucleus and cytoplasm (Fig 4A). LPA increased HIF1α expression in the nucleus as we have shown previously [26]. Similarly, MIF immunofluorescence signal in the nucleus was markedly increased in response to LPA, suggesting that the interaction of MIF with HIF1α occurs in the nucleus. This finding was further validated by co-immunoprecipitation of HIF1α and MIF. To this end, we performed immunoprecipitation of MIF using a limited amount of anti-MIF antibody (0.5 μg) under an assumption that the amount of antibody is the limiting factor so that similar amounts of MIF will be immunoprecipitated from all samples. Accordingly, the amounts of immunoprecipitated MIF did not differ substantially in all samples (Fig 4B, MIF light exposure). Consistent with the confocal immunofluorescence microscopy, the interaction of MIF with HIF1α under basal conditions was marginal. In contrast, LPA significantly augmented their interaction, which was ablated by knockdown of LPA_2_ (Fig 4B). Control immunopreciptation using rabbit IgG did not pull down HIF1α (S1A Fig), demonstrating the specificity of the interaction. Although these results imply that LPA facilitates the interaction between HIF1α and MIF, this could be due to increased expression of these two proteins by LPA. To address this possibility, we expressed MIF-2xFLAG and HA-HIF1α in HCT116 cells. Treating cells with LPA enhanced co-immunoprecipitation of MIF-2xFLAG with HA-HIF1α and conversely ISO-1 mitigated their interaction (Fig 4C). These results suggest that, in addition to inducing HIF1α and MIF expression, LPA facilitates the interaction between these two proteins.

### CSN5 stabilizes HIF and HIF1α

**Fig 5:**
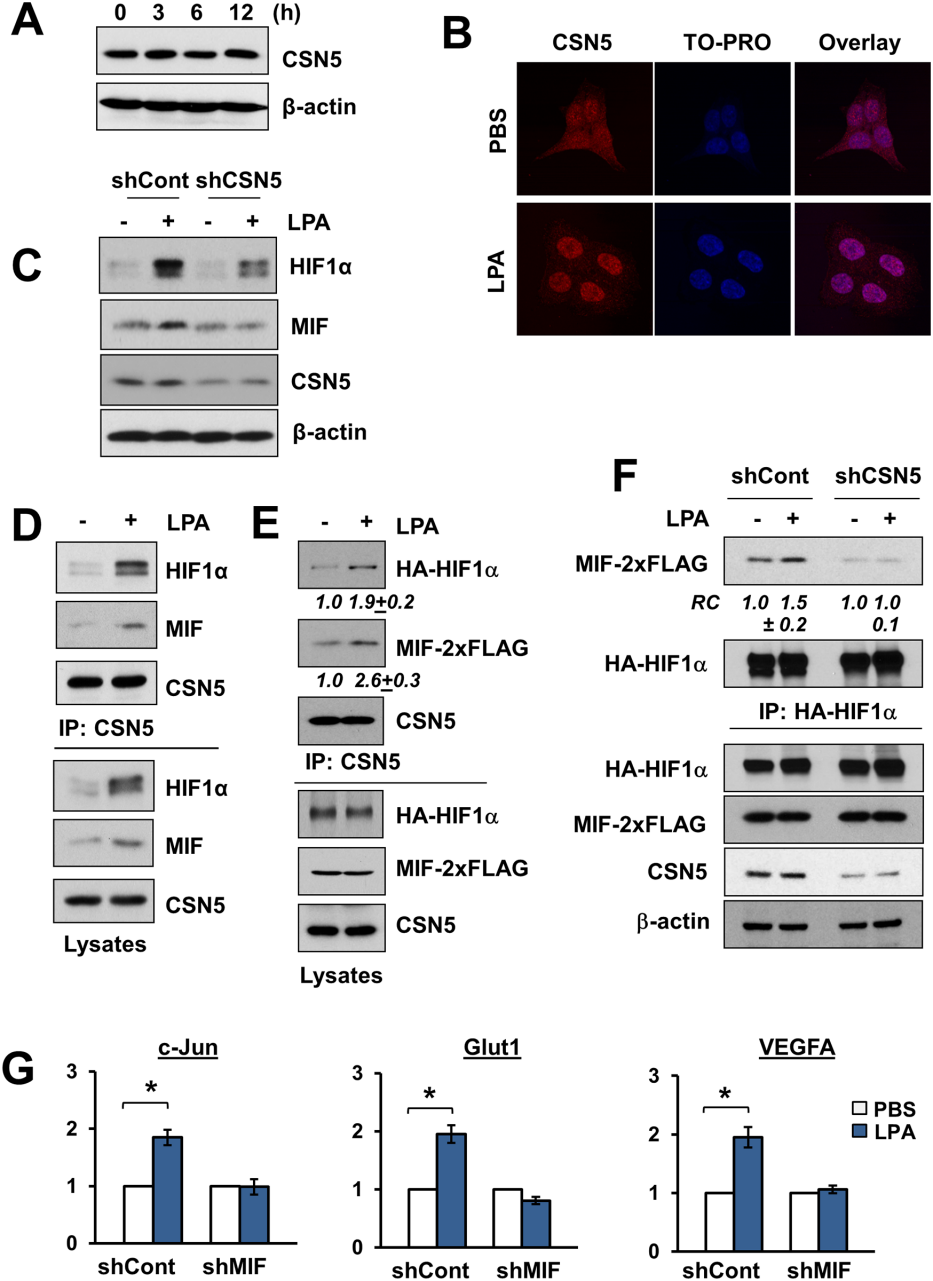
MIF and HIF1α interaction requires CSN5. **(A)** CSN5 expression in HCT116 cells treated with LPA is shown. **(B)** Cellular localization of CSN5 (red) in cells treated with LPA was determined by immunofluorescence confocal microscopy. TO-PRO was used for nuclear counterstaining (blue). **(C)** Induction of HIF1α and MIF by LPA in cells with or without CSN5 knockdown was determined. **(D)** Co-immunoprecipitation of HIF1α and MIF with CSN5 (left panels) from HCT116 cell lysates is shown. Lysates from LPA or PBS treated cells were immunoprecipitated with anti-CSN5 antibody, followed by Western blotting with anti-HIF1α or anti-MIF antibody. Lower panels show HIF1α, MIF, and CSN5 expression in cell lysates. **(E)** HA-HIF1α and MIF-2xFLAG were expressed in HCT116 cells, and co-immunoprecipitation of HA-HIF1α and MIF-2xFLAG with CSN5 was determined. Lower panels show HA-HIF1α, MIF-2xFLAG, and CSN5 expression in cell lysates. **(F)** MIF-2xFLAG was co-immunoprecipitated with HA-HIF1α from cells infected with shCont or shCSN5. Lower panels show protein expression in cell lysates. Representative blots from three independent experiments are shown is all studies. **(G)** HCT116/shCont and HCT116/shMIF cells were treated with LPA and mRNA levels (mean ± SEM) of c-Jun, Glut1, and VEGFA were determined by qRT-PCR. n = 3. *, *p* < 0.05 compared with untreated cells.

Internally, MIF binds the CSN5 subunit of the COP9 signalosome (CSN). Intracellular MIF sequesters CSN5 to block AP-1 transcriptional factor activity [32]. In addition, HIF1α is stabilized under aerobic conditions via its interaction with CSN5 [33]. Hence, we investigated whether CSN5 is involved in the regulation of MIF by LPA. LPA did not alter the expression level of CSN5 (Fig 5A), but LPA increased CSN5 abundance in the nucleus (Fig 5B), indicating that LPA promotes nuclear translocation of CSN5. We also found that depletion of CSN5 decreased LPA-induced HIF1α and MIF protein expression (Fig 5C). Because CSN5 is known to interact with MIF under hypoxic conditions [30], we determined whether the same interaction occurs in cell treated with LPA. As shown in Fig 5D, LPA increased the efficiency of MIF co-immunoprecipitating with CSN5, implying that LPA facilitates their interaction. In addition, the association of CSN5 with HIF1α was also augmented by LPA in line with a previous study that overexpression of CSN5 in some tumors stabilizes HIF1α by preventing HIF1α hydroxylation [34]. To circumvent the possibility that the increased interaction was due to increased HIF1α and MIF expression under LPA-treated conditions, we assessed the interaction of CSN5 with HIF1α or MIF in cells exogenously expressing HA-HIF1α and MIF-2xFLAG. LPA stimulated co-immunoprecipitation of HA-HIF1α and MIF-2xFLAG with CSN5 (Fig 5E), but not with control IgG (S1B Fig), indicating that the improved interaction was not due to increased protein expression. We showed in Fig 4B that MIF and HIF1α co-immunoprecipitated in LPA-treated cells, but it was unclear if this interaction was direct or mediated by CSN5. To address this question, we performed co-immunoprecipitation of MIF-2xFLAG and HA-HIF1α. Depletion of CSN5 markedly decreased the association of HA-HIF1α and MIF-2xFLAG under basal conditions and blocked LPA-induced interaction (Fig 5F). In order to correlate the HIF1α-MIF association with the transcriptional activity of HIF1α, we determined mRNA levels of HIF1α target genes. Fig 5G shows that LPA stimulated c-Jun, glucose transporter 1 (Glut1), and vascular endothelial growth factor A (VEGFA) mRNA levels. Knockdown of MIF abolished LPA-mediated induction of these genes, demonstrating that the presence of MIF is critical for the transcriptional activity of HIF1α. Together, these data indicate that LPA enhances the HIF1α-MIF association in a CSN5-dependent mechanism.

## Discussion

MIF is a pro-inflammatory mediator whose expression is closely linked to the process of oncogenic transformation and tumor growth [18–20]. In many cases, MIF is constitutively expressed and stored in intracellular pools, and does not require de novo protein synthesis like other cytokines [1]. Previous studies showed that LPA induced MIF in the mouse colon 26 cell line and loss of LPA_2_ decreased MIF expression in mouse intestine [19, 25], but how LPA regulates MIF has not been determined. Expression of MIF often correlates with the state of hypoxia, although MIF expression is not dependent on hypoxia or HIF1α in MCF-7 breast cancer cells [35–37]. In this study, we have shown that LPA increases MIF expression in colon cancer cells and its regulation is dependent on the transcription activation by HIF1α. Our previous study showed that LPA-induced HIF1α activation involves suppression of wild type p53 [26]. In addition, LPA induces Krüppel-like factor 5 (KLF5) expression, which displaces p53 from the HIF1α promoter [26]. MIF regulation by LPA mirrors that of HIF1α, as such MIF induction was observed only in wild type p53 expressing HCT116 and LoVo cells. It has been shown that MIF bypasses p53-mediated growth arrest and apoptosis [20, 38]. However, the temporal sequence of p53 suppression occurring at an earlier time-point (~ 1 h after addition of LPA) compared with MIF induction (> 3h) makes it unlikely that MIF is involved in p53 regulation [26]. [35–37][39][40]

The current study shows that LPA increases cellular MIF expression and secretion of MIF into the extracellular medium. Knockdown or inhibition of MIF attenuated LPA-mediated cell proliferation, supporting the functional importance of MIF. ISO-1 is an inhibitor of tautomerase activity of MIF that has been localized crystallographically to the protein’s N-terminal substrate binding site [41]. Tautomerase-null MIF retains its ability to bind CD74 and CSN5 and the tumor forming capacity, indicating that the tautomerase activity of MIF is dispensable for its transformational function [42]. On the other hand, others have shown that the tautomerase activity of MIF is essential for tumor growth and metastasis [43], and the relationship between the tautomerase activity of MIF and its biological functions remains controversial. Although ISO-1 inhibits the tautomerase activity of MIF, it is noteworthy that ISO-1 can inhibit MIF binding to its receptor with an IC50 of only 10 μM [41]. Therefore, it is difficult to distinguish whether the inhibitory effect of ISO-1 was through inhibition of the tautomerase activity of MIF or blockade of MIF binding to surface receptors., The effect of extracellular rMIF stimulating cell proliferation appears to support receptor-mediated effect of MIF. However, MIF can readily be internalized [32], and again we cannot exclude the possibility that rMIF supports cell proliferation via a receptor-independent mechanism. Although MIF is necessary for LPA-mediated colon cancer cell proliferation, how MIF facilitates this effect is not clear. Previously, we showed that HIF1α depletion reduced the rate of HCT116 cells proliferation [26]. Since MIF expression reciprocally regulates HIF1α protein expression, we speculate that MIF indirectly impacts cell proliferation through HIF1α transcriptional activity. Interestingly, we observed that neither MIF depletion nor ISO-1 altered the basal proliferation of HCT116 cells. However, the absence of effect is consistent with a previous study that MIF knockdown did not affect the basal RhoA and focal adhesion kinase activities [19] and that MIF-deficiency in mice did not alter basal physiological functions unless the animals were challenged [40, 44, 45].

HIF1α is rapidly degraded by the von Hippel-Lindau tumor suppressor protein (pVHL)-dependent ubiquitination in the presence of oxygen [46]. However, how LPA maintains HIF1α expression despite the presence of oxygen remains unclear. We found that MIF depletion was refractory to LPA-induced HIF1α expression. MIF did not alter HIF1α transcription, but inhibition of proteasomes by MG-132 stabilized HIF1α even in cells with MIF depleted, indicating that MIF maintains the stability of HIF1α protein. We also found that CSN5 plays a pivotal role in maintenace of HIF1α stability in LPA-treated cells. CSN5 is a component of the COP9 signalosome that participates in diverse cellular and developmental processes [47]. CSN5 binds the C-terminal oxygen-dependent degradation domain of HIF1α and pVHL to protect HIF1α from aerobic degradation [34]. The relationship between MIF and CSN5 is complex. CSN5 rescues cells from a cyclin dependent kinase inhibitor p27^Kip1^, and MIF interacts with CSN5 to antagonize CSN5-dependent rescue of fibroblasts from growth arrest [32]. Lue et al. [48] reported that CSN5 functions as a molecular link that retains MIF in the intracellular pools, indicating that CSN5 negatively regulates autocrine MIF activity. However, under hypoxic conditions MIF enhances CSN5 binding to HIF1α, thereby stimulating HIF1α stability and amplifying hypoxic responses [30].

In the current study, we provide convincing evidence that MIF physically and functionally interacts with CSN5 to stabilize HIF1α, placing CSN5 at the center of the tripartite interaction. LPA enhanced the interaction of CSN5 with MIF or HIF1α. [30]LPA stimulated the interaction between HIF1α and MIF as evidenced by co-immunoprecipitation and confocal immunofluorescence microscopic co-localization. This interaction appears indirect through CSN5 since depletion of CSN5 attenuated the association of exogenous HIF1α and MIF, placing CSN5 at the center of the tripartite interaction. This dynamic effect of LPA differs from hypoxia which does not affect the MIF-CSN5 interaction in MIA-PaCa-2 cells [30]. How LPA facilitates the interaction of CSN5 with HIF1α and MIF is not known. Regulatory mechanism of CSN5 or MIF is not well known except the effects on their expression levels. In the current study, we showed that LPA stimulated the interaction of CSN5 with exogenously expressed MIF-2xFLAG and HA-HIF1α (Fig 5F). This result implies that LPA may post-translationally regulate their interaction, but because MIF is known to form homo-multimers [9], we cannot completely forgo the possibility that endogenous MIF induced by LPA contributes to the increased interaction. Nonetheless, we observed that LPA enhanced the expression of MIF and CSN5 in the nucleus. Hence, one way that LPA promotes their interaction is by clustering the three proteins in the nucleus.

In summary, we show that LPA induces MIF expression via a HIF1α-dependent mechanism. The stabilization of HIF1α requires CSN5 and MIF, with CSN5 forming the bridge between HIF1α and MIF. However, future studies are needed to reveal the molecular details of their interaction.

## Supporting Information

S1 Fig **(A)** HCT116 cells treated with LPA (+) or PBS (-) were lysed and immunoprecipitated with rabbit IgG, followed by immunoblotting for HIF1α, MIF, or CSN5. **(B)** A representative figure of negative co-immunoprecipitation of HA-HIF1α and MIF-2xFLAG is shown.
(PDF)
