# Supplementary material for: HIF1α-Induced by Lysophosphatidic Acid Is Stabilized via Interaction with MIF and CSN5
Source: PLoS One. 2015 Sep 9;10(9):e0137513. doi: 10.1371/journal.pone.0137513 (PMC4564097; doi:10.1371/journal.pone.0137513)
Supplement: S1 Fig — (A) HCT116 cells treated with LPA (+) or PBS (-) were lysed and immunoprecipitated with rabbit IgG, followed by immunoblotting for HIF1α, MIF, or CSN5. (B) A representative figure of negative co-immunoprecipitation of HA-HIF1α and MIF-2xFLAG is shown. (PDF) [file pone.0137513.s001.pdf]

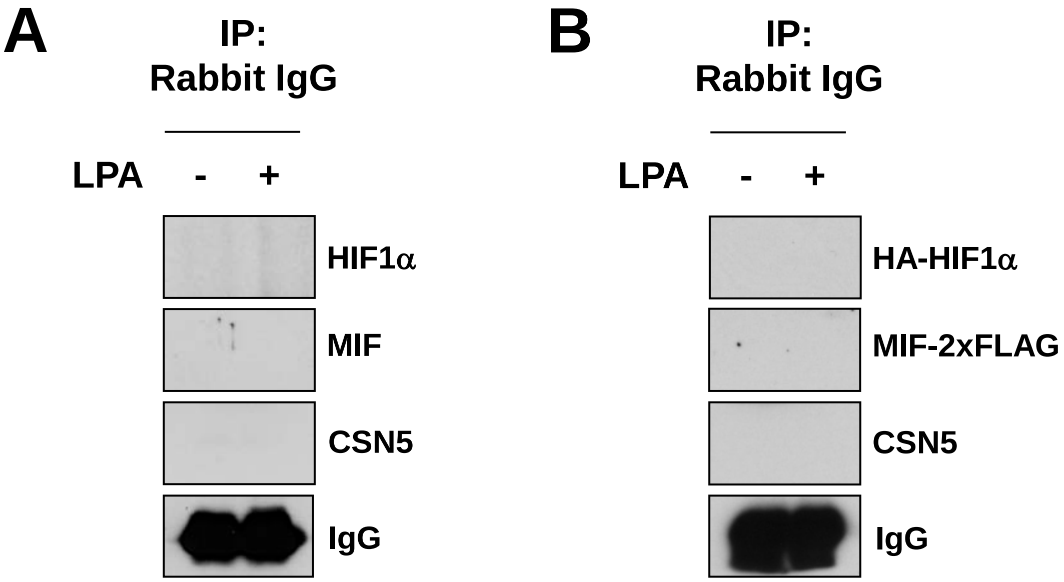

Supplemental Figure 1

(A) HCT116 cells treated with LPA (+) or PBS (-) were lysed and immunoprecipitated with rabbit IgG, followed by immunoblotting for HIF1α, MIF, or CSN5.
(B) A representative figure of negative co-immunoprecipitation of HA-HIF1α and MIF-2xFLAG is shown.
